# Recent advances in understanding the role of Cdk1 in the Spindle Assembly Checkpoint

**DOI:** 10.12688/f1000research.21185.1

**Published:** 2020-01-28

**Authors:** Angela Flavia Serpico, Domenico Grieco

**Affiliations:** 1 CEINGE Biotecnologie Avanzate, Naples, 80145, Italy; 2 DMMBM, University of Naples "Federico II", Naples, 80131, Italy; 3 Department of Pharmacy, University of Naples "Federico II", Naples, 80131, Italy

**Keywords:** Cdk1, APC/C, MCC, Cdc20, CCAN, Mps1, Mad1, Mad2, Bub1, spindle assembly checkpoint, SAC.

## Abstract

The goal of mitosis is to form two daughter cells each containing one copy of each mother cell chromosome, replicated in the previous S phase. To achieve this, sister chromatids held together back-to-back at their primary constriction, the centromere, have to interact with microtubules of the mitotic spindle so that each chromatid takes connections with microtubules emanating from opposite spindle poles (we will refer to this condition as bipolar attachment). Only once all replicated chromosomes have reached bipolar attachments can sister chromatids lose cohesion with each other, at the onset of anaphase, and move toward opposite spindle poles, being segregated into what will soon become the daughter cell nucleus. Prevention of errors in chromosome segregation is granted by a safeguard mechanism called Spindle Assembly Checkpoint (SAC). Until all chromosomes are bipolarly oriented at the equator of the mitotic spindle, the SAC prevents loss of sister chromatid cohesion, thus anaphase onset, and maintains the mitotic state by inhibiting inactivation of the major M phase promoting kinase, the cyclin B-cdk1 complex (Cdk1). Here, we review recent mechanistic insights about the circuitry that links Cdk1 to the SAC to ensure correct achievement of the goal of mitosis.

## Introduction

Maintenance of genome stability through cell generations is a crucial feature that grants health to cells, organs and organisms. In humans, genome instability is causally linked to pathological outcomes such as cancer, degenerative disorders and physical and mental retardation ^1– 3^. Cells have developed several mechanisms to surveil that each step required for cell division is healthy and thoroughly completed before passing to the next one. This is achieved through mechanisms called cell cycle checkpoints ^4– 8^. If cells experience DNA damage or sense that DNA replication or assembly of the mitotic spindle is incomplete, checkpoint mechanisms halt cell cycle progression to repair damage or complete previous cell cycle stages before moving forward in their division process. If repair or completion is frustrated, then healthy checkpoints promote cell death ^9– 13^. This short review will be focused on recent advancements in the mechanistic understanding of the Spindle Assembly Checkpoint (SAC), the checkpoint that prevents formation of cells with an abnormal chromosome number by delaying mitosis exit until bipolar attachment of all replicated chromosomes ^14^.

## Progression through mitosis: a cycle of Cdk1 activation/inactivation

Progression through mitosis is granted by a wave of cyclin B-cdk1 complex (Cdk1) activity ^15, 16^. Cdk1 is activated at the onset of mitosis by reversal of inhibitory phosphorylations of the cdk1 moiety at threonine 14 and tyrosine 15. These phosphorylations, operated by the Myt1 and Wee1 kinases, allow accumulation of enough inactive Cdk1, during S phase and G _2_, to rapidly induce mitosis upon their reversal ^17, 18^. Dephosphorylation and activation of Cdk1 are granted by the dual-specificity phosphatase Cdc25 ^19^. Upon initial activation, Cdk1 phosphorylates and inhibits Myt1 and Wee1 while it phosphorylates and further activates Cdc25; this way, Cdk1 promotes positive feedback loops for its own activation ^20– 22^. For mitosis onset, Cdk1 activity also represses major phosphatase activities (like that of PP1 and PP2A) that otherwise would antagonize Cdk1 action. The catalytic activity of PP1 is directly inhibited by Cdk1-dependent phosphorylation, while the activity of PP2A in which B55 is the holoenzyme regulatory subunit, PP2A-B55, is kept inhibited in mitosis by the aid of Greatwall kinase (Gwl). Gwl is stimulated by Cdk1 and phosphorylates Ensa/Arpp19, two small molecules, transforming them into potent PP2A-B55 inhibitors ^22^.

Inactivation of Cdk1 at the end of mitosis instead depends on the ubiquitin-dependent degradation of cyclin B ^14, 23– 25^. This is initiated by the ubiquitin ligase Anaphase Promoting Complex/Cyclosome (APC/C) in association with its coactivator Cdc20. APC/C ^Cdc20^ also promotes the degradation of securin, an inhibitor of separase, the protease that cleaves the protein bridge that holds sister chromatid centromeres together ^14, 26– 28^. This way, the onset of anaphase and Cdk1 inactivation are tightly coupled by this irreversible degradative mechanism. Initial evidence indicated that APC/C ^Cdc20^ activity required Cdk1-dependent phosphorylation; recently, the APC/C members that are directly phosphorylated by Cdk1 were identified ^29– 33^. Thus, Cdk1 is also promoting a negative feedback for its own inactivation. Nevertheless, final APC/C ^Cdc20^ activation is under the control of the SAC, which inhibits APC/C ^Cdc20^ until bipolar attachment of all replicated chromosomes ^14^.

## Mps1 and the SAC, in brief

**Figure 1.**
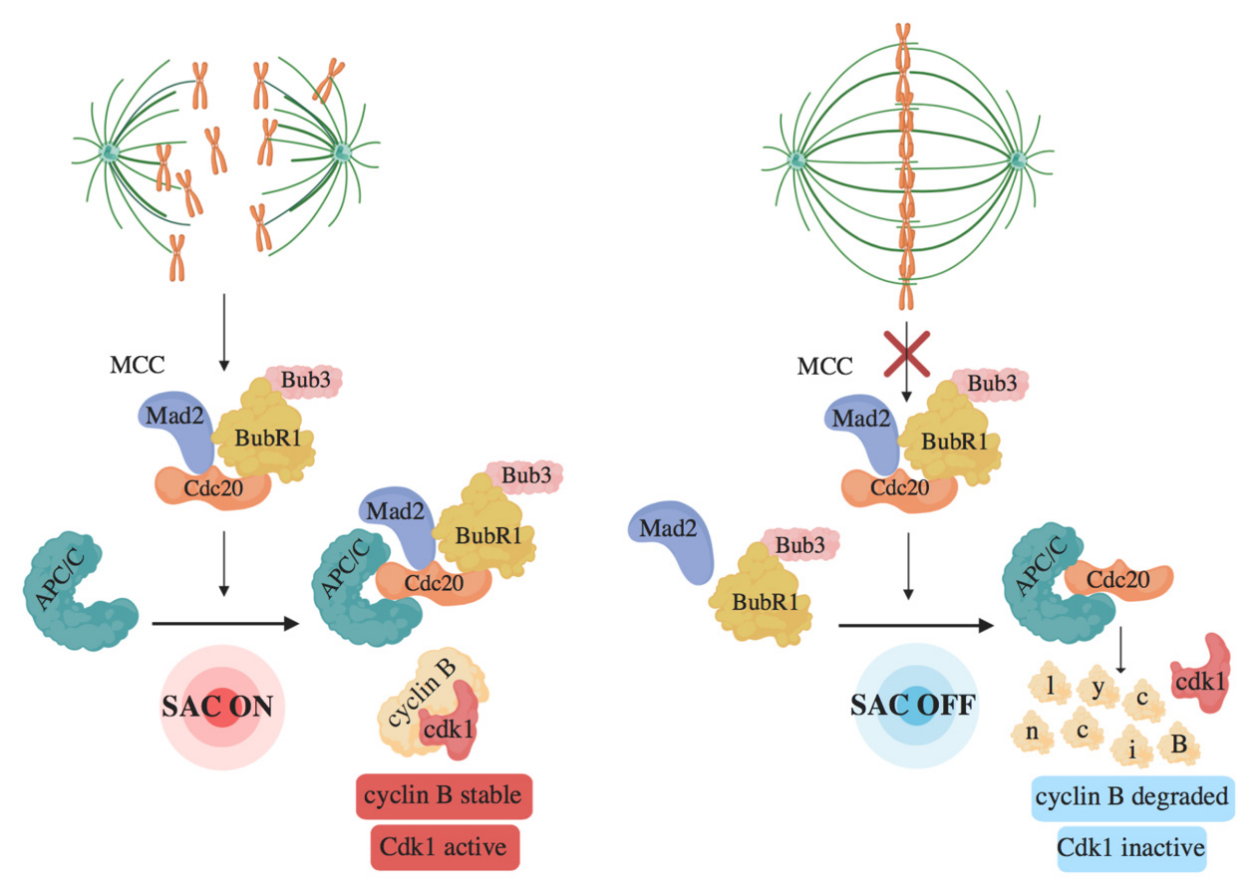
Unattached or incorrectly attached chromosomes promote formation of the Mitotic Checkpoint Complex (MCC). Until bipolar spindle assembly, the MCC, composed of Mad2, BubR1-Bub3, and Cdc20, forms, binds, and blocks APC/C action (SAC ON). Upon bipolar spindle assembly, MCC is dismantled and MCC-free Cdc20 activates APC/C (SAC OFF).

**Figure 2.**
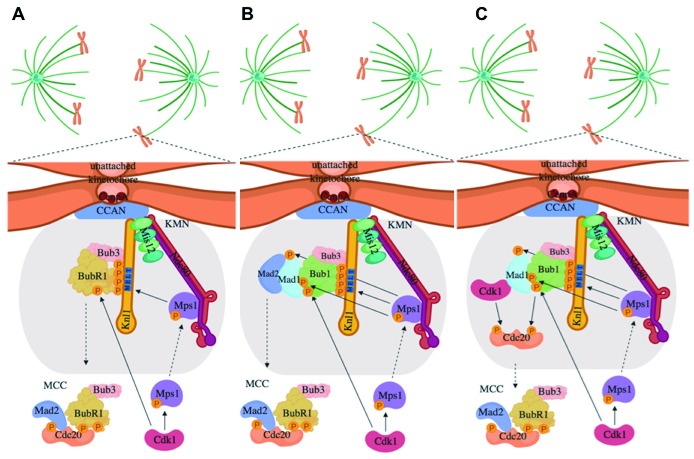
Paths to Mitotic Checkpoint Complex (MCC) formation. Cdk1 phosphorylation of Mps1 helps kinetochore recruitment of Mps1 to ( **A**) recruit BubR1-Bub3 complex for its incorporation into MCC, ( **B**) recruit Bub1-Bub3 for Mad1-Mad2 docking and Mad2 incorporation into MCC, and ( **C**) recruit Bub1-Bub3 for Mad1-Cdk1 docking for Bub1- and Cdk1-dependent phosphorylation of Cdc20 and incorporation into MCC.

The SAC inhibits APC/C ^Cdc20^ activation by forming a diffusible Mitotic Checkpoint Complex (MCC), composed of the proteins Mad2, Bub3, BubR1, and Cdc20 itself, in which Cdc20 is restrained from activating APC/C ^14, 34– 37^. MCC forms at unattached kinetochores, proteinaceous centromeric structures deputed to interact with spindle microtubules and permit chromosome segregation ( Figure 1) ^14^. MCC formation requires the action of crucial SAC kinases like Plk1, Aurora B, and Mps1 ^38– 40^. These kinases also have important roles in correcting faulty chromosome–microtubule interactions to promote correct, end-on, bipolar chromosome–microtubule attachments ^41^. Here, however, we will primarily review recent advancements in the regulation of Mps1 in SAC control and its dependence on Cdk1 activity. Mps1 binds unattached kinetochores where it phosphorylates SAC proteins and activates them and then gets released from kinetochores upon stable microtubule binding, perhaps by competition mechanisms ^42– 46^. The bridge deputed to connect centromeres to microtubules is called the KMN network and is composed by the Knl1 complex, the Mis12 complex, and the Ndc80 complex ^46– 50^. The KMN, in the outer kinetochore, interacts with the inner kinetochore Constitutive Centromere Associated Network (CCAN), a protein network that assembles onto Cenp-A nucleosomes, a histone H3 variant found at centromeric nucleosomes ^51– 53^. Mps1 localizes at unattached kinetochores primarily by interacting with the Ndc80 complex ^54^. At kinetochores, Mps1 phosphorylates the “MELT” repeats of Knl1, promoting kinetochore recruitment of the BubR1-Bub3 and Bub1-Bub3 complexes ( Figure 2), while Knl1 dephosphorylation by PP1 appears involved in SAC silencing ^43, 55– 58^. Mps1 also phosphorylates Bub1, further promoting kinetochore recruitment of Mad1, another crucial SAC protein needed for the activation of Mad2 ^59– 61^. Mad1 recruitment at kinetochores is also facilitated by the Rod-Zwilch-ZW10 (RZZ) complex ^62^. In addition, phosphorylation of Mad1 by Mps1 helps the Mad1-dependent conversion of Mad2 into the functional conformation required to inhibit Cdc20 in the MCC ^59, 60^. Thus, Mps1 is a crucial effector of the SAC mechanism by promoting MCC formation ( Figure 2).

## Cdk1 and the SAC

The observation that APC/C activity was promoted by Cdk1-dependent phosphorylation, while APC/C activation was inhibited by the SAC until spindle assembly, reinforced the idea that checkpoint mechanisms would oppose the forward trend of the basic cell cycle engine ^4^. However, in 2003, a few independent observations, from yeast and vertebrates, changed this view by showing that Cdk1 was instrumental to the SAC action ^63– 65^. Indeed, in yeast, SAC-defective cdk1 mutants were described and Bub1 was shown to be phosphorylated by Cdk1 for SAC proficiency ^63, 64^. In the Xenopus egg extract system and in human somatic cells, Cdk1 activity was revealed to be required to sustain SAC-dependent arrest and the ability of MCC members to block the APC/C ^65, 66^. Cdk1-dependent phosphorylation of Cdc20 appeared to have a role in reducing Cdc20 affinity for APC/C while increasing that for other MCC proteins ^65– 68^. Thus, Cdk1, the cell cycle engine, though paving the way for its own inactivation by phosphorylating APC/C, was instrumental for the checkpoint SAC that would block APC/C activation until correct spindle assembly ^66^. These observations also helped to explain why the SAC does not get reactivated at the onset of anaphase, when loss of chromatid cohesion causes loss of kinetochore tension, a condition that would have activated the SAC at earlier stages ^69, 70^. This was shown to be due to the concomitant reduction of Cdk1 because of the mentioned coupling of anaphase onset with degradation of cyclin B ^69, 70^. A few years later, the notion that Cdk1 was required for the SAC function was reinforced by the findings that, in the Xenopus egg extract system, Mps1 was phosphorylated by Cdk1 and that this phosphorylation substantially helped Mps1 activity in its fundamental role for the SAC ^71^.

Very recently, through careful biochemical dissection, important observations have described in closer detail how Cdk1 is an integral part of the SAC mechanisms ^72, 73^. Indeed, it has been shown that kinetochore localization of Mps1, in human cells, greatly depends on direct phosphorylation by Cdk1; thus, Cdk1 controls activity and localization of Mps1 ^72^. Mps1, in turn, helps kinetochore localization of Cdk1 ^73– 76^. As mentioned earlier, by phosphorylating Knl1, Mps1 creates a docking site for kinetochore localization of Bub1, and cooperative Cdk1- and Mps1-dependent phosphorylations of Bub1 are required to recruit Mad1 at kinetochores ^42, 43, 50, 59, 77^. Kinetochore localization of Mad1 is crucial for its ability to convert Mad2 in the effective form that incorporates into the MCC ^61^. However, it has also recently been shown that Mad1 stably interacts with Cdk1 and that Mps1, through kinetochore recruitment of Mad1, in turn, promotes kinetochore localization of Cdk1 ( Figure 2) ^72, 73^. At kinetochores, Cdk1 may further phosphorylate other substrates to sustain the SAC like Cdc20 or BubR1 and possibly also help error correction and SAC resolution by favoring BubR1 interaction with the protein phosphatase PP2A-B56 ^65, 78– 80^. Recent evidence also indicated how the indirect downregulation of the protein phosphatase PP2A-B55 activity by Cdk1 is instrumental for the SAC-promoting action of Cdk1 itself ^72, 81^. In addition, it should be noted that kinetochore localization of Mps1 is favored by the activity of Aurora B, perhaps by phosphorylating members of the Ndc80 complex ^42^. However, centromere localization of Aurora B depends on other components of the Chromosomal Passenger Complex (CPC), composed of survivin, borealin, INCENP, and Aurora B itself, and Cdk1 activity is required, directly and indirectly, for CPC centromeric localization ^82– 84^. Thus, even by mastering CPC localization, Cdk1 affects Mps1 and is fundamental for SAC action.

## Concluding remarks and further questions

The recent advancements, reviewed here, in the mechanisms of mitotic exit and in particular in how Cdk1 mechanistically serves the SAC, suggest that Cdk1 is an integral part of the SAC system. Thus, perhaps the cell cycle engine, Cdk1, and the checkpoint, SAC, are not to be viewed any longer as separate mechanisms but rather as integrated systems that ensure correct execution of complex biological tasks. Important hints have also been recently provided on how the SAC can be silenced, such as on priming mechanisms for protein phosphatases that would reverse SAC-activating phosphorylations upon bipolar chromosome attachments, in addition to the notion that the MCC itself undergoes proteasome-dependent turnover for rapid SAC silencing ^79, 84– 88^. Nevertheless, major phosphatases like PP1 and PP2A are directly or indirectly inhibited by Cdk1 activity ^22^. Thus, it is still unclear whether chromosome attachment and kinetochore tension are sufficient to dislodge kinases and let phosphatases take the upper hand for SAC silencing or whether these conditions also affect the activity of crucial SAC kinases ^40^. Based on our previous observations, we hypothesize in this regard that Cdk1 activity could be locally downregulated by non-proteolytic means upon bipolar chromosome attachment and that this would lead to SAC silencing ^89– 92^. If this were true, a proteolysis-independent negative control of Cdk1 would be required for SAC silencing, ahead of and for final, proteolysis-dependent, Cdk1 inactivation and mitotic exit.

## Abbreviations

APC/C, Anaphase Promoting Complex/Cyclosome; Cdk1, cyclin B-cdk1 complex; CPC, Chromosomal Passenger Complex; Gwl, Greatwall kinase; CCAN, Constitutive Centromere Associated Network; KNM, Knl1 complex, Ndc80 complex, Mis12 complex; MCC, Mitotic Checkpoint Complex; SAC, Spindle Assembly Checkpoint

## References

[1] Taylor AMR Rothblum-Oviatt C Ellis NA: Chromosome instability syndromes. *Nat Rev Dis Primers.* 2019;5(1):64. 10.1038/s41572-019-0113-0 31537806 PMC10617425

[2] Bach DH Zhang W Sood AK: Chromosomal Instability in Tumor Initiation and Development. *Cancer Res.* 2019;79(16):3995–4002. 10.1158/0008-5472.CAN-18-3235 31350294 PMC7694409

[3] de Wolf B Kops GJPL: Kinetochore Malfunction in Human Pathologies. *Adv Exp Med Biol.* 2017;1002:69–91. 10.1007/978-3-319-57127-0_4 28600783

[4] Hartwell LH Weinert TA: Checkpoints: controls that ensure the order of cell cycle events. *Science.* 1989;246(4930):629–34. 10.1126/science.2683079 2683079

[5] Murray AW: Creative blocks: cell-cycle checkpoints and feedback controls. *Nature.* 1992;359(6396):599–604. 10.1038/359599a0 1406993

[6] Elledge SJ: Cell cycle checkpoints: preventing an identity crisis. *Science.* 1996;274(5293):1664–72. 10.1126/science.274.5293.1664 8939848

[7] Nurse P: Checkpoint pathways come of age. *Cell.* 1997;91(7):865–7. 10.1016/s0092-8674(00)80476-6 9428508

[8] Musacchio A: Spindle assembly checkpoint: the third decade. *Philos Trans R Soc Lond B Biol Sci.* 2011;366(1584):3595–604. 10.1098/rstb.2011.0072 22084386 PMC3203455

[9] Hartwell LH Kastan MB: Cell cycle control and cancer. *Science.* 1994;266(5192):1821–8. 10.1126/science.7997877 7997877

[10] Sorger PK Dobles M Tournebize R: Coupling cell division and cell death to microtubule dynamics. *Curr Opin Cell Biol.* 1997;9(6):807–14. 10.1016/s0955-0674(97)80081-6 9425345

[11] Jacotot E Ferri KF Kroemer G: Apoptosis and cell cycle: distinct checkpoints with overlapping upstream control. *Pathol Biol (Paris).* 2000;48(3):271–9. 10858959

[12] Clarke PR Allan LA: Cell-cycle control in the face of damage--a matter of life or death. *Trends Cell Biol.* 2009;19(3):89–98. 10.1016/j.tcb.2008.12.003 19168356

[13] Krenning L van den Berg J Medema RH: Life or Death after a Break: What Determines the Choice? *Mol Cell.* 2019;76(2):346–58. 10.1016/j.molcel.2019.08.023 31561953

[14] Musacchio A Salmon ED: The spindle-assembly checkpoint in space and time. *Nat Rev Mol Cell Biol.* 2007;8(5):379–93. 10.1038/nrm2163 17426725

[15] Nurse P: Universal control mechanism regulating onset of M-phase. *Nature.* 1990;344(6266):503–8. 10.1038/344503a0 2138713

[16] Minshull J Pines J Golsteyn R: The role of cyclin synthesis, modification and destruction in the control of cell division. *J Cell Sci.* 1989;12:77–97. 10.1242/jcs.1989.supplement_12.8 2534558

[17] Atherton-Fessler S Hannig G Piwnica-Worms H: Reversible tyrosine phosphorylation and cell cycle control. *Semin Cell Biol.* 1993;4(6):433–42. 10.1006/scel.1993.1051 8305682

[18] Deibler RW Kirschner MW: Quantitative reconstitution of mitotic CDK1 activation in somatic cell extracts. *Mol Cell.* 2010;37(6):753–67. 10.1016/j.molcel.2010.02.023 20347419 PMC2882237

[19] Perdiguero E Nebreda AR: Regulation of Cdc25C activity during the meiotic G2/M transition. *Cell Cycle.* 2014;3(6):733–7. 10.4161/cc.3.6.906 15136768

[20] Kapuy O He E López-Avilés S: System-level feedbacks control cell cycle progression. *FEBS Lett.* 2009;583(24):3992–8. 10.1016/j.febslet.2009.08.023 19703449 PMC3811919

[21] Domingo-Sananes MR Kapuy O Hunt T: Switches and latches: A biochemical tug-of-war between the kinases and phosphatases that control mitosis. *Philos Trans R Soc Lond B Biol Sci.* 2011;366(1584):3584–94. 10.1098/rstb.2011.0087 22084385 PMC3203464

[22] Hunt T: On the regulation of protein phosphatase 2A and its role in controlling entry into and exit from mitosis. *Adv Biol Regul.* 2013;53(2):173–8. 10.1016/j.jbior.2013.04.001 23672858

[23] Evans T Rosenthal ET Youngblom J: Cyclin: a protein specified by maternal mRNA in sea urchin eggs that is destroyed at each cleavage division. *Cell.* 1983;33(2):389–96. 10.1016/0092-8674(83)90420-8 6134587

[24] Murray AW Solomon MJ Kirschner MW: The role of cyclin synthesis and degradation in the control of maturation promoting factor activity. *Nature.* 1989;339(6222):280–6. 10.1038/339280a0 2566918

[25] King RW Peters JM Tugendreich S: A 20S complex containing CDC27 and CDC16 catalyzes the mitosis-specific conjugation of ubiquitin to cyclin B. *Cell.* 1995;81(2):279–88. 10.1016/0092-8674(95)90338-0 7736580

[26] Michaelis C Ciosk R Nasmyth K: Cohesins: chromosomal proteins that prevent premature separation of sister chromatids. *Cell.* 1997;91(1):35–45. 10.1016/s0092-8674(01)80007-6 9335333

[27] Ciosk R Zachariae W Michaelis C: An ESP1/PDS1 complex regulates loss of sister chromatid cohesion at the metaphase to anaphase transition in yeast. *Cell.* 1998;93(6):1067–76. 10.1016/s0092-8674(00)81211-8 9635435

[28] Lu D Girard JR Li W: Quantitative framework for ordered degradation of APC/C substrates. *BMC Biol.* 2015;13:96. 10.1186/s12915-015-0205-6 26573515 PMC4647693

[29] Shteinberg M Protopopov Y Listovsky T: Phosphorylation of the cyclosome is required for its stimulation by Fizzy/cdc20. *Biochem Biophys Res Commun.* 1999;260(1):193–8. 10.1006/bbrc.1999.0884 10381365

[30] Rudner AD Murray AW: Phosphorylation by Cdc28 activates the Cdc20-dependent activity of the anaphase-promoting complex. *J Cell Biol.* 2000;149(7):1377–90. 10.1083/jcb.149.7.1377 10871279 PMC2175139

[31] Zhang S Chang L Alfieri C: Molecular mechanism of APC/C activation by mitotic phosphorylation. *Nature.* 2016;533(7602):260–4. 10.1038/nature17973 27120157 PMC4878669

[32] Fujimitsu K Grimaldi M Yamano H: Cyclin-dependent kinase 1-dependent activation of APC/C ubiquitin ligase. *Science.* 2016;352(6289):1121–4. 10.1126/science.aad3925 27103671

[33] Qiao R Weissmann F Yamaguchi M: Mechanism of APC/C CDC20 activation by mitotic phosphorylation. *Proc Natl Acad Sci U S A.* 2016;113(19):E2570–E2578. 10.1073/pnas.1604929113 27114510 PMC4868491

[34] Sudakin V Chan GK Yen TJ: Checkpoint inhibition of the APC/C in HeLa cells is mediated by a complex of BUBR1, BUB3, CDC20, and MAD2. *J Cell Biol.* 2001;154(5):925–36. 10.1083/jcb.200102093 11535616 PMC2196190

[35] Díaz-Martínez LA Yu H: Running on a treadmill: dynamic inhibition of APC/C by the spindle checkpoint. *Cell Div.* 2007;2: 23. 10.1186/1747-1028-2-23 17650307 PMC1947974

[36] Izawa D Pines J: The mitotic checkpoint complex binds a second CDC20 to inhibit active APC/C. *Nature.* 2015;517(7536):631–4. 10.1038/nature13911 25383541 PMC4312099

[37] Alfieri C Chang L Zhang Z: Molecular basis of APC/C regulation by the spindle assembly checkpoint. *Nature.* 2016;536(7617):431–6. 10.1038/nature19083 27509861 PMC5019344

[38] Nigg EA: Mitotic kinases as regulators of cell division and its checkpoints. *Nat Rev Mol Cell Biol.* 2001;2(1):21–32. 10.1038/35048096 11413462

[39] Suijkerbuijk SJ Kops GJ: Preventing aneuploidy: the contribution of mitotic checkpoint proteins. *Biochim Biophys Acta.* 2008;1786(1):24–31. 10.1016/j.bbcan.2008.04.001 18472014

[40] Saurin AT: Kinase and Phosphatase Cross-Talk at the Kinetochore. *Front Cell Dev Biol.* 2018;6:62. 10.3389/fcell.2018.00062 29971233 PMC6018199

[41] Manic G Corradi F Sistigu A: Molecular Regulation of the Spindle Assembly Checkpoint by Kinases and Phosphatases. *Int Rev Cell Mol Biol.* 2017;328:105–61. 10.1016/bs.ircmb.2016.08.004 28069132

[42] Santaguida S Tighe A D'Alise AM: Dissecting the role of MPS1 in chromosome biorientation and the spindle checkpoint through the small molecule inhibitor reversine. *J Cell Biol.* 2010;190(1):73–87. 10.1083/jcb.201001036 20624901 PMC2911657

[43] Yamagishi Y Yang CH Tanno Y: MPS1/Mph1 phosphorylates the kinetochore protein KNL1/Spc7 to recruit SAC components. *Nat Cell Biol.* 2012;14(7):746–52. 10.1038/ncb2515 22660415

[44] Hiruma Y Sacristan C Pachis ST: CELL DIVISION CYCLE. Competition between MPS1 and microtubules at kinetochores regulates spindle checkpoint signaling. *Science.* 2015;348(6240):1264–7. 10.1126/science.aaa4055 26068855

[45] Ji Z Gao H Yu H: CELL DIVISION CYCLE. Kinetochore attachment sensed by competitive Mps1 and microtubule binding to Ndc80C. *Science.* 2015;348(6240):1260–4. 10.1126/science.aaa4029 26068854

[46] Aravamudhan P Goldfarb AA Joglekar AP: The kinetochore encodes a mechanical switch to disrupt spindle assembly checkpoint signalling. *Nat Cell Biol.* 2015;17(7):868–79. 10.1038/ncb3179 26053220 PMC4630029

[47] Varma D Wan X Cheerambathur D: Spindle assembly checkpoint proteins are positioned close to core microtubule attachment sites at kinetochores. *J Cell Biol.* 2013;202(5):735–46. 10.1083/jcb.201304197 23979716 PMC3760617

[48] Petrovic A Keller J Liu Y: Structure of the MIS12 Complex and Molecular Basis of Its Interaction with CENP-C at Human Kinetochores. *Cell.* 2016;167(4):1028–1040.e15. 10.1016/j.cell.2016.10.005 27881301 PMC5101189

[49] Umbreit NT Gestaut DR Tien JF: The Ndc80 kinetochore complex directly modulates microtubule dynamics. *Proc Natl Acad Sci U S A.* 2012;109(40):16113–8. 10.1073/pnas.1209615109 22908300 PMC3479545

[50] Varma D Salmon ED: The KMN protein network--chief conductors of the kinetochore orchestra. *J Cell Sci.* 2012;125(Pt 24):5927–36. 10.1242/jcs.093724 23418356 PMC3585512

[51] Hori T Amano M Suzuki A: CCAN makes multiple contacts with centromeric DNA to provide distinct pathways to the outer kinetochore. *Cell.* 2008;135(6):1039–52. 10.1016/j.cell.2008.10.019 19070575

[52] Screpanti E de Antoni A Alushin GM: Direct binding of Cenp-C to the Mis12 complex joins the inner and outer kinetochore. *Curr Biol.* 2011;21(5):391–8. 10.1016/j.cub.2010.12.039 21353556 PMC3074538

[53] Hori T Shang WH Takeuchi K: The CCAN recruits CENP-A to the centromere and forms the structural core for kinetochore assembly. *J Cell Biol.* 2013;200(1):45–60. 10.1083/jcb.201210106 23277427 PMC3542802

[54] Stucke VM Baumann C Nigg EA: Kinetochore localization and microtubule interaction of the human spindle checkpoint kinase Mps1. *Chromosoma.* 2004;113(1):1–15. 10.1007/s00412-004-0288-2 15235793

[55] Shepperd LA Meadows JC Sochaj AM: Phosphodependent recruitment of Bub1 and Bub3 to Spc7/KNL1 by Mph1 kinase maintains the spindle checkpoint. *Curr Biol.* 2012;22(10):891–9. 10.1016/j.cub.2012.03.051 22521786 PMC3780767

[56] Primorac I Weir JR Chiroli E: Bub3 reads phosphorylated MELT repeats to promote spindle assembly checkpoint signaling. *eLife.* 2013;2:e01030. 10.7554/eLife.01030 24066227 PMC3779320

[57] Zhang G Mendez BL Sedgwick GG: Two functionally distinct kinetochore pools of BubR1 ensure accurate chromosome segregation. *Nat Commun.* 2016;7: 12256. 10.1038/ncomms12256 27457023 PMC4963475

[58] Rosenberg JS Cross FR Funabiki H: KNL1/Spc105 recruits PP1 to silence the spindle assembly checkpoint. *Curr Biol.* 2011;21(11):942–7. 10.1016/j.cub.2011.04.011 21640906 PMC3109435

[59] Ji Z Gao H Jia L: A sequential multi-target Mps1 phosphorylation cascade promotes spindle checkpoint signaling. *eLife.* 2017;6: pii: e22513. 10.7554/eLife.22513 28072388 PMC5268738

[60] Faesen AC Thanasoula M Maffini S: Basis of catalytic assembly of the mitotic checkpoint complex. *Nature.* 2017;542(7642):498–502. 10.1038/nature21384 28102834 PMC5448665

[61] Sironi L Melixetian M Faretta M: Mad2 binding to Mad1 and Cdc20, rather than oligomerization, is required for the spindle checkpoint. *EMBO J.* 2001;20(22):6371–82. 10.1093/emboj/20.22.6371 11707408 PMC125308

[62] Caldas GV Lynch TR Anderson R: The RZZ complex requires the N-terminus of KNL1 to mediate optimal Mad1 kinetochore localization in human cells. *Open Biol.* 2015;5(11): pii: 150160. 10.1098/rsob.150160 26581576 PMC4680571

[63] Kitazono AA Garza DA Kron SJ: Mutations in the yeast cyclin-dependent kinase Cdc28 reveal a role in the spindle assembly checkpoint. *Mol Genet Genomics.* 2003;269(5):672–84. 10.1007/s00438-003-0870-y 12827501

[64] Yamaguchi S Decottignies A Nurse P: Function of Cdc2p-dependent Bub1p phosphorylation and Bub1p kinase activity in the mitotic and meiotic spindle checkpoint. *EMBO J.* 2003;22(5):1075–87. 10.1093/emboj/cdg100 12606573 PMC150333

[65] D'Angiolella V Mari C Nocera D: The spindle checkpoint requires cyclin-dependent kinase activity. *Genes Dev.* 2003;17(20):2520–5. 10.1101/gad.267603 14561775 PMC218146

[66] D'Angiolella V Grieco D: Attach first, then detach: a role for cyclin B-dependent kinase 1 in coordinating proteolysis with spindle assembly. *Cell Cycle.* 2004;3(2):132–3. 10.4161/cc.3.2.664 14712073

[67] Hein JB Hertz EPT Garvanska DH: Distinct kinetics of serine and threonine dephosphorylation are essential for mitosis. *Nat Cell Biol.* 2017;19(12):1433–40. 10.1038/ncb3634 29084198

[68] Labit H Fujimitsu K Bayin NS: Dephosphorylation of Cdc20 is required for its C-box-dependent activation of the APC/C. *EMBO J.* 2012;31(15):3351–62. 10.1038/emboj.2012.168 22713866 PMC3411074

[69] Rattani A Vinod PK Godwin J: Dependency of the spindle assembly checkpoint on Cdk1 renders the anaphase transition irreversible. *Curr Biol.* 2014;24(6):630–7. 10.1016/j.cub.2014.01.033 24583015 PMC3969274

[70] Vázquez-Novelle MD Sansregret L Dick AE: Cdk1 inactivation terminates mitotic checkpoint surveillance and stabilizes kinetochore attachments in anaphase. *Curr Biol.* 2014;24(6):638–45. 10.1016/j.cub.2014.01.034 24583019 PMC3969148

[71] Morin V Prieto S Melines S: CDK-dependent potentiation of MPS1 kinase activity is essential to the mitotic checkpoint. *Curr Biol.* 2012;22(4):289–95. 10.1016/j.cub.2011.12.048 22245000

[72] Hayward D Alfonso-Pérez T Cundell MJ: CDK1-CCNB1 creates a spindle checkpoint-permissive state by enabling MPS1 kinetochore localization. *J Cell Biol.* 2019;218(4):1182–99. 10.1083/jcb.201808014 30674582 PMC6446832

[73] Alfonso-Pérez T Hayward D Holder J: MAD1-dependent recruitment of CDK1-CCNB1 to kinetochores promotes spindle checkpoint signaling. *J Cell Biol.* 2019;218(4):1108–17. 10.1083/jcb.201808015 30674583 PMC6446853

[74] Pfaff KL King RW: Determinants of human cyclin B1 association with mitotic chromosomes. *PLoS One.* 2013;8(3):e59169. 10.1371/journal.pone.0059169 23505570 PMC3594322

[75] Allan LA Reis M Liu Y: Cyclin B1 scaffolds MAD1 at the corona to activate the spindle assembly checkpoint. *BioRxiv.* 2019; 726224. 10.1101/726224

[76] Jackman M Marcozzi C Pardo M: Cyclin B1-Cdk1 binding to MAD1 links nuclear pore disassembly to chromosomal stability. *BioRxiv.* 2019; 701474. 10.1101/701474

[77] Zhang G Kruse T López-Méndez B: Bub1 positions Mad1 close to KNL1 MELT repeats to promote checkpoint signalling. *Nat Commun.* 2017;8: 15822. 10.1038/ncomms15822 28604727 PMC5472792

[78] Wong OK Fang G: Cdk1 phosphorylation of BubR1 controls spindle checkpoint arrest and Plk1-mediated formation of the 3F3/2 epitope. *J Cell Biol.* 2007;179(4):611–7. 10.1083/jcb.200708044 17998400 PMC2080899

[79] Kruse T Zhang G Larsen MS: Direct binding between BubR1 and B56-PP2A phosphatase complexes regulate mitotic progression. *J Cell Sci.* 2013;126(Pt 5):1086–92. 10.1242/jcs.122481 23345399

[80] Hayward D Bancroft J Mangat D: Checkpoint signaling and error correction require regulation of the MPS1 T-loop by PP2A-B56. *J Cell Biol.* 2019;218(10):3188–99. 10.1083/jcb.201905026 31511308 PMC6781431

[81] Diril MK Bisteau X Kitagawa M: Loss of the Greatwall Kinase Weakens the Spindle Assembly Checkpoint. *PLoS Genet.* 2016;12(9):e1006310. 10.1371/journal.pgen.1006310 27631493 PMC5025047

[82] Ruchaud S Carmena M Earnshaw WC: Chromosomal passengers: conducting cell division. *Nat Rev Mol Cell Biol.* 2007;8(10):798–812. 10.1038/nrm2257 17848966

[83] Tsukahara T Tanno Y Watanabe Y: Phosphorylation of the CPC by Cdk1 promotes chromosome bi-orientation. *Nature.* 2010;467(7316):719–23. 10.1038/nature09390 20739936

[84] Feng H Raasholm M Moosmann A: Switching of INCENP paralogs controls transitions in mitotic chromosomal passenger complex functions. *Cell Cycle.* 2019;18(17):2006–25. 10.1080/15384101.2019.1634954 31306061 PMC6681789

[85] Nijenhuis W Vallardi G Teixeira A: Negative feedback at kinetochores underlies a responsive spindle checkpoint signal. *Nat Cell Biol.* 2014;16(12):1257–64. 10.1038/ncb3065 25402682 PMC6485516

[86] Ge S Skaar JR Pagano M: APC/C- and Mad2-mediated degradation of Cdc20 during spindle checkpoint activation. *Cell Cycle.* 2014;8(1):167–71. 10.4161/cc.8.1.7606 19098431 PMC2703714

[87] Visconti R Palazzo L Grieco D: Requirement for proteolysis in spindle assembly checkpoint silencing. *Cell Cycle.* 2014;9(3):564–9. 10.4161/cc.9.3.10581 20081372

[88] Varetti G Guida C Santaguida S: Homeostatic control of mitotic arrest. *Mol Cell.* 2011;44(5):710–20. 10.1016/j.molcel.2011.11.014 22152475

[89] D'Angiolella V Palazzo L Santarpia C: Role for non-proteolytic control of M-phase-promoting factor activity at M-phase exit. *PLoS One.* 2007;2(2):e247. 10.1371/journal.pone.0000247 17327911 PMC1803016

[90] Visconti R Palazzo L Della Monica R: Fcp1-dependent dephosphorylation is required for M-phase-promoting factor inactivation at mitosis exit. *Nat Commun.* 2012;3: 894. 10.1038/ncomms1886 22692537 PMC3621406

[91] Visconti R Della Monica R Palazzo L: The Fcp1-Wee1-Cdk1 axis affects spindle assembly checkpoint robustness and sensitivity to antimicrotubule cancer drugs. *Cell Death Differ.* 2015;22(9):1551–60. 10.1038/cdd.2015.13 25744022 PMC4532778

[92] Della Monica R Visconti R Cervone N: Fcp1 phosphatase controls Greatwall kinase to promote PP2A-B55 activation and mitotic progression. *eLife.* 2015;4: pii: e10399. 10.7554/eLife.10399 26653855 PMC4749544

